# Report of a Case of Gonorrheal Arthritis Treated with Antigonococcic Serum

**Published:** 1909-03

**Authors:** Thomas W. Parsche

**Affiliations:** Chicago


### Report of a Case of Gonorrheal Arthritis Treated with Antigonococcic Serum

By THOMAS W. PARSCHE, M. D., Chicago.

Mr. A. J. D., age, 24 years, single, stenographer, was first seen at my office January 30, 1908. He stated that he had contracted gonorrhea on December 26, 1907, and had been treated for the condition by a physician, by the injection method, the acute discharge disappearing in eleven days after the onset. On January 15, 1908, he complained of pains in the left knee and consulted his physician. In three days the knee became so swollen and inflamed that he was unable to bend it and had to use crutches. The physician told him that he had gonorrheal rheumatism of the left knee joint and gave him ointments to apply, but the condition resisted treatment.

*Status presens.*—He complains of a great deal of pain in the left knee joint, has chills and fever, night sweats, poor appetite, constipation, a urethral discharge and a very badly swollen knee joint. He was unable to flex or extend the left leg. Upon physical examination I found that he had a temperature of 102, pulse 112, tongue coated white, chest negative, abdomen negative, discharge from urethra, blood count 12,000. The left knee was greatly enlarged and swollen, measuring in circumference 27 inches at the patella, that of the right knee being 19 inches. The patella was immobile, fluctuation present in the patella and the tibial bursæ and it was impossible to manipulate the joint; flexion and tension were gone. The inflammation extended down to the middle third of the leg. Microscopical examination of the urethral smear showed gonococci present. I advised him to go to St. Joseph's Hospital which he did the following morning.

*Treatment.*—A plaster cast was applied to the left leg and thigh, the patient was put to bed and placed on a milk diet. Urethral injections of 25 per cent. argyrol were given daily and the internal medication consisted of strontium salicylate grs. 5, every two hours, and oil of santal wood E. I., M 15, three times a day. Codeine phosphate was given at night for sleep. On the second day after the cast was applied the pain became so intense in the knee joint that the cast was cut, forming anterior and posterior splints. The knee was painted with 50 per cent. tincture iodine and again immobilised. At the end of the first week a new cast was applied with a window over the knee joint and hot boric fomentations were applied. During this time the patient was kept on milk diet. At no time was he free from pain which was so severe that morphia had to be given for relief.

During the next two weeks I applied the Bier hyperemic treatment twice daily. The cupping became so painful and, instead of reducing the inflammatory condition, it seemed to aggravate the condition so, that I had to discontinue it. The evening temperature during this time was averaging 99 degrees F. The urethral discharge had ceased and there were no shreds in the urine.

Dr. Robert H. Herbst very kindly saw the case and recommended the use of antigonococcic serum of Parke, Davis & Co. On February 17, 1908, the patient was injected with 2 cc. of the serum, but there was no reaction. Again, on the following day, he was injected with a like amount, but no reaction followed. After further injection of 4 cc. there was a rise in the temperature of two degrees and he developed urticaria at the point of injection. The dose of the serum was raised to 6 cc., and the reaction was very prompt with an edema of the scrotum. The point selected for the injection was the abdomen. At the end of the first week the pain had disappeared and the swelling was subsiding.

Hot magnesium sulphate fomentations were applied to the knee and 6 cc. of the serum was injected, every second day. On March 7, the swelling had nearly disappeared and he was able to partially flex and extend the knee. The light diet was continued and he was given no red meats. He left the hospital March 14, on crutches and could bear the weight of his body on the left leg.

The serum injection were reduced to 4 cc. every three days until May 12, when he was given 2 cc. weekly until the 1st of July.

*Final Results.*—July 1, there was perfect motion of the knee joint the swelling had disappeared together with all the pain. Measurements, over the patella, 20 inches. He is able to flex the leg completely on the thigh.

1321 SHERIDAN ROAD.

---

### SYMPTOMATOLOGY OF TUBERCULOSIS OF THE LARYNX.

GEORGE FETTEROLF of Philadelphia, Pa., states the desirability of an early diagnosis in tuberculous laryngitis. This may be made by considering the following symptoms. Changes in the voice, from slight hoarseness to complete aphonia; cough that is harsh and causes little expectoration in proportion to is amount and severity; sputum that is cast off in dry masses in the morning; blood generally comes from the lungs; pain is universal in tuberculous laryngitis, and is often very severe; dysphagia, odynphagia, and dyspnea are sometimes seen.—*Medical Record,* January 23. 1909.

